# The process of developing evidence-based guidance in medicine and public health: a qualitative study of views from the inside

**DOI:** 10.1186/1748-5908-8-101

**Published:** 2013-09-04

**Authors:** Lou Atkins, Jonathan A Smith, Michael P Kelly, Susan Michie

**Affiliations:** 1 Research Department of Clinical, Education and Health Psychology, Centre for Outcomes Research and Effectiveness (CORE), University College London, 1-19 Torrington Place, London WC1E 7HB, UK; 2 Department of Psychological Sciences, Birkbeck University of London, Malet St., London WC1E 7HX, UK; 3 Centre for Public Health, National Institute for Health and Care Excellence, 10, Spring Gardens, London, SW1A 2BU, And Institute of Public Health, University of Cambridge, Cambridge CB2 0SR, UK

**Keywords:** Advisory groups, Content analysis, Evidence, NICE guidelines, Qualitative

## Abstract

**Background:**

There has been significant investment in developing guidelines to improve clinical and public health practice. Though much is known about the processes of evidence synthesis and evidence-based guidelines implementation, we know little about how evidence presented to advisory groups is interpreted and used to form practice recommendations or what happens where evidence is lacking. This study investigates how members of advisory groups of NICE (National Institute of Health and Clinical Excellence) conceptualize evidence and experience the process.

**Methods:**

Members of three advisory groups for acute physical, mental and public health were interviewed at the beginning and end of the life of the group. Seventeen were interviewed at both time points; five were interviewed just once at time one; and 17 were interviewed only once after guidance completion. Using thematic and content analysis, interview transcripts were analysed to identify the main themes.

**Results:**

Three themes were identified:

1. What is the task? Different members conceptualized the task differently; some emphasized the importance of evidence at the top of the quality hierarchy while others emphasized the importance of personal experience.

2. Who gets heard? Managing the diversity of opinion and vested interests was a challenge for the groups; service users were valued and as was the importance of fostering good working relationships between group members.

3. What is the process? Group members valued debate and recognized the need to marshal discussion; most members were satisfied with the process and output.

**Conclusions:**

Evidence doesn’t form recommendations on its own, but requires human judgement. Diversity of opinion within advisory groups was seen as key to making well-informed judgments relevant to forming recommendations. However, that diversity can bring tensions in the evaluation of evidence and its translation into practice recommendations.

## Background

Before the rise of evidence-based medicine (EBM), and evidence-based practice (EBP) more generally, clinicians made decisions about clinical care and directors of public health made decisions about public health policies and measures. The recognition that such decisions should be evidence-based raised the issue as to how evidence should be identified, synthesized and translated into recommendations. Organizations, such as the National Institute for Health and Care Excellence (NICE)^a^ in the United Kingdom (UK), were established to do this. The model developed by NICE was explicitly based on the principles of deliberative democracy [1,2]. Such organizations set up advisory groups that bring together key groups of people: those with the technical expertise to synthesize research evidence, those with clinical and public health expertise and those with relevant ‘lay’ or service user experience. The latter two groups are necessary to interpret the research evidence within the clinical and social contexts within which the recommendations are to be applied. While this has brought strengths in terms of plurality of experiences, perspectives, and backgrounds to inform evidence-based recommendations, it has also brought challenges that need to be recognized and managed to optimize process and outcome.

Guideline development has been described as involving two processes: technical (evidence synthesis) and social (recommendation formation) [3]. Much is known about the technical processes of evidence synthesis (as exemplified by the Cochrane Collaboration and its methodology group) and about the implementation of evidence-based guidelines (as described in the journal *Implementation Science*). In contrast, little is known about the social process of how evidence presented to advisory groups is interpreted and used to form practice recommendations and the impact of diversity within advisory groups on this process. The functioning of such groups is a ‘black box’ between evidence and ‘evidence-based’ recommendations [4-8].

There has been little primary research into how evidence is translated into guideline recommendations. Reports on guidelines have relied on contributions from social psychology to explain group processes and decision making or opinion pieces on methods for synthesizing views of advisory group members opinions [9]. One observational study of two advisory groups reported that discussions could be coded into four domains: science, practice, politics, and process [10].

What direct evidence exists suggests that producing guidelines is not merely a simple process of translating evidence into recommendations and evidence may not always be the main influence on recommendation formation [11]. An experimental study of nominal group technique in clinical guideline development reported that clinical experience and beliefs were more influential than research evidence recommendations [12]. Similarly, a systematic review of factors influencing judgment in formal consensus development methods reported that ratings of the appropriateness of a procedure were positively related to group members’ experience of performing that procedure [13]. The same review reported the effect of the range of specialisms within an advisory group on recommendations; multidisciplinary groups were observed to make more conservative recommendations than unidisciplinary groups.

The impact of factors such as the professional status of group members on advisory group dynamics has also been investigated. An observational study of guideline development for primary care by a multi-disciplinary panel reported greater discussion between higher status than lower status professionals [14].

Evidence from an observational study of recommendations for coronary diagnostic procedures suggests that interpretations of the same evidence differ between groups as only moderate agreement between two different panels of clinicians forming recommendations on the same clinical issue was observed [15].

A synthesis of studies of service user involvement in clinical practice guideline development recorded key barriers and facilitators to service user involvement [16]. Most common barriers to service user involvement included discrepancies between the perspectives of health professionals and those of service users; a lack of familiarity with scientific terminology; under-representation of service users compared with other advisory group members; and difficulties recruiting service users to advisory groups. Key facilitators of service user involvement were training and support: email and phone support between meetings and support from the Chair, in particular, within meetings to encourage contributions to discussion and working in small groups.

No study to our knowledge has examined the processes of how evidence is translated into recommendations through the lens of those serving on such groups.

The aim of this study is to investigate how evidence is translated into recommendations from the perspective of NICE guidance groups. The specific research questions are:

1. Who is perceived as having most influence and does this lead to the dominance of particular approaches?

2. What strategies are used in considering evidence and in formulating recommendations?

3. What beliefs about the purpose and nature of evidence and recommendations may explain these strategies?

4. What are group members’ views about the quality of group processes and outcomes?

This paper reports interview data from a purposive sample of members of three guidance advisory groups developing recommendations for clinical practice and public health for NICE. Members were interviewed twice, at the beginning and end of the life of the group. The data are analysed qualitatively and quantitatively. The study forms part of a wider program of research investigating the processes by which EBP recommendations are made [6].

## Methods

### Sample

A purposive sample of 39 members of three NICE advisory groups was drawn (acute physical health, mental health and public health). The sample represented different constituencies within the groups: three Chairs, 16 healthcare professionals (including 4 clinical psychologists, four psychiatrists, two nurses, two hospital doctors, one clinical adviser, one pharmacist, one general practitioner (GP), one paramedic), nine technical team members (four health economists, two project managers, one systematic reviewer, one research fellow, one information scientist), seven service users^b^ (including two community members on public health program development group (PGD)), two professional members (lecturer and civil servant) on public health PGD and two service providers (from public health organizations) were interviewed.

### Procedure

The study received ethical approval from the Research Ethics Committee of the UCL Psychology Department to approach and obtain consent from advisory group members at the beginning of the guidance development process (ref: 0819/001). Advisory group members were provided with an information sheet that gave details about the aims of the project and what their participation would entail, written consent was obtained before interviews were conducted. Face-to-face or telephone interviews, depending on preference, were conducted in a location convenient to the participant. The interviewers were University-based researchers (RD and JC) independent of NICE.

The aim was to interview each participant twice, once at the beginning of the guidance development process after one or two meetings (time1) and again at the end of the process when guidance was being, or had been, finalised (time 2). Logistical constraints resulted in 17 (44%) of the 39 participants being interviewed twice.

### Interview schedule

The interview consisted of open-ended questions to elicit group members’:

1. expectations and experiences of the advisory group;

2. their perceived role in the group;

3. their own and others’ conceptualisations of evidence and of the purpose of guideline recommendations;

4. significant incidents of disagreement or conflict and influences on decision making;

5. their contribution to the advisory group.

In the second interview participants were also asked:

1. to reflect on their experiences as an advisory group member;

2. whether they thought the process had changed their views;

3. how satisfied they were with the finished guideline.

Interviews lasted 30 to 45 minutes (Additional file 1).

### Data collection

Thirty-nine interviews were conducted totalling 75 hours of recorded material. These included: seventeen repeated interviews (six mental health, six acute physical health, five public health); five first interviews only (one mental health, two acute physical health, two public health), 17 second interviews only (11 mental health, six acute physical health). Roles of advisory group members interviewed over two time points are summarised in Table 1. Digital recordings were transcribed and data were anonymized as far as possible.

**Table 1:** Advisory group member role summary

**Advisory group**	**Role**
Mental health	Chair (healthcare professional), service user, healthcare professional 1, healthcare professional 2, systematic reviewer*, health economist*
Acute physical health	Chair (healthcare professional), service user, service user representative (working for a patient group), healthcare professional 1, healthcare professional 2, health economist*
Public health	Chair, professional member 1, professional member 2, community member 1, community member 2

Participants are uniquely identifiable by the roles listed in Table 1.

*Systematic reviewers and health economists are commissioned by NICE and have no role in guideline formation.

### Data analysis

#### Methodological approach

A thematic analysis was conducted to identify recurrent themes in the data. This was followed by a content analysis of the number of utterances to provide quantitative data to contextualize identified themes.

#### Analysis

Repeated interviews (interviews for which a before and after interview had been conducted) were analysed for one advisory group in the first instance (the mental health advisory group). Two researchers (LA and SM) independently analysed the transcripts to identify themes. LA listed emergent themes after reading a pair of transcripts and discussed these themes with SM. Data related to main themes were extracted into a table. Raw data in the form of participants’ quotes reflecting the themes were entered into a spread sheet, grouped according to themes and type of advisory group, participant, and interview timing. This allowed specific comparisons of advisory group, participant role, and perspectives over time.

Common links between themes were noted and formed the basis of superordinate and subordinate themes. SM and LA discussed the two sets of analyses, which were generally similar, and agreed the main findings. General reflections and thoughts on the themes were also recorded.

Transcripts from the second advisory group were analysed within the framework of the first advisory group looking for confirmation and disconfirmation of earlier themes but also allowing the emergence of new themes. This process was repeated for transcripts from the third advisory group.

Following the analysis of repeat interviews, single non-repeat interviews were analysed to check data saturation, *i.e.*, that no new themes were identified and that no emergent themes were contradicted by this second set of data.

LA and SM produced a first draft narrative account based on identified themes and reflections of the researchers. This was reviewed by JS and MK and the thematic structure was refined and the narrative account improved. To protect anonymity and for ease of interpretation, members are referred to in terms of their role (Chair, healthcare professional, service user, or role in the technical team such as systematic reviewer or health economist) and the guidance group to which they belong (mental health, acute physical health, or public health).

A content analysis was conducted to extract quantitative data from transcripts. The number of times group members made a comment that supported identified subordinate themes was recorded. These data were and tabulated and are presented broken down by advisory group and group member role.

## Results

Theme saturation was achieved and three super-ordinate themes related to the four main research questions were identified:

1. What is the task—how did advisory group members conceptualise what they were charged with doing (related to research question—What beliefs about the purpose and nature of evidence and recommendations may explain strategies to consider evidence and form recommendations)?

2. Who gets heard—who contributed to discussions and/or influenced decision making (related to research question—who has most influence and does this lead to the dominance of particular approaches)?

3. What is the process—what were group members’ experiences and evaluations of the advisory group process (related to research questions—What strategies are used in considering evidence and in formulating recommendations? What are GDG members’ views about the quality of GDG process and outcome)?

Views expressed by advisory group members who had been interviewed twice on the guidance development process were broadly consistent over time; there were no significant changes in their perceptions between the two interviews.

### What is the task?

There were contrasting views on issues fundamental to the guidance development process—what recommendations should be used for and about the nature of evidence and how it should be considered.

### What is the purpose of recommendations?

The range of opinions about the purpose of guidance recommendations highlighted the tension between recommendations being aspirational, serving as a long-term lever of change and being immediately implementable into current practice, considering financial and practical feasibility. Examples of aspirational purpose are:

‘If you only do what’s actually achievable, or easily implemented, um, you’re not putting any pressure on the system to change. And it’s quite useful to use guidelines as a lever of change’. (Healthcare professional 2, MH1^c^)

Examples of immediate implementability are:

‘I don’t think aspirational, because I think that just gives an excuse not to do anything’. (Service user, MH2)

A content analysis (Table 2) revealed an almost even split of statements supporting guidance as aspirational versus immediately implementable. This theme was discussed mostly in the mental and acute physical health advisory groups with only two statements about this issue in the public health group.

**Table 2:** Purpose of recommendations

**Theme**	**Total**	**Advisory group**			**Members**			
		**Acute physical health**	**Mental health**	**Public health**	**Chair**	**Health profs.**	**Service users**	**Technical team**
Aspirational	22	8	14	0	2	7	7	6
Immediately implementable	24	7	15	2	6	3	5	8

### What is evidence?

Two themes emerged from responses to questions about the nature of evidence: how group members defined evidence and how they appraised it.

Group members defined evidence in two ways, a ‘scientific’ definition based on peer-review literature presented to the group and a broader definition based on professional and lay experience of members and drawing on a wider range of sources.

There was awareness that different views were held about what constitutes evidence:

‘I would imagine there would be a range of views, from those people who think that the only kind of evidence worth looking at …^d^ is meta-analysis … through to those people who…are sceptical about how well some of those trials defend themselves against threats to external validity’. (Healthcare professional 2, MH1)

Those subscribing to the ‘scientific’ definition viewed EBM as the gold standard of evidence that everyone should agree with:

‘Well, evidence is, in the setting of NICE guidelines really refers to high-quality evidence, which is evidence in randomized control trials’. (Chair, MH1)

Examples of the broad definition of evidence are:

‘I think expert opinion, patient opinion’s all evidence actually, if I’m pushed on it’. (Healthcare professional 1, AH1^e^)

‘There’s a number of different types of evidence I suppose. … gold standard, as it’s called, being the randomized control trial published in a peer review paper … but … evidence I suppose goes right the way down to individual experience as well’. (Service user, MH1)

Those who held a broad view of evidence thought there was the potential for exaggerating the significance of small findings with a narrow, ‘scientific’ conceptualisation of evidence:

‘People take a single small trial and over-interpret it. … there’s a sort of pretence that the more you systematize it … the more you get rid of these issues. But in fact you don’t, you just hide them. In some ways you make the problem worse’. (Healthcare professional, AH1)

Table 3 contains frequency data indicating that the mental health advisory group spent more time debating what evidence is than did the acute physical or public health groups. The advisory group chairs spoke more about ‘scientific evidence’ whereas service users spent more time talking more about evidence ‘of experience’, which reflects their respective professional backgrounds and experience.

**Table 3:** ‘Scientific’ evidence versus evidence ‘of experience’

**Theme**	**Total**	**Advisory group**			**Members**			
		**Acute physical health**	**Mental health**	**Public health**	**Chair**	**Health profs.**	**Service users**	**Technical team**
‘Scientific’ evidence	29	6	14	9	6	15	3	5
Evidence ‘of experience’	24	8	10	6	1	11	9	3

In cases where ’scientific’ evidence was of poor quality, sparse, or contradictory, group members offered different solutions. Difficulties were anticipated in reaching agreement on recommendation based on weak evidence:

‘The bits of the guideline where the data is much less clear … it’s not randomized control trials with clear outcomes, it’s always the hardest bit in a way because you’re … relying on the groups to come to some kind of consensus expert opinion, which is always trickier’. (Systematic reviewer, MH2)

The imperative to provide a recommendation drove members to look for evidence other than randomized controlled trials. Some regarded this very much as ‘second best’:

‘Where we don’t have RCTs…we have to downgrade our standards, and we look for other kind of evidence’. (Health economist, MH2)

‘The whole point about NICE is to try and look at what the best evidence is and make recommendations on that basis. So coming up with a consensus is, I think, a bit of a second best’. (Service User Representative, AH2)

Reliance only on expert opinion was viewed by some as ‘pontification’ and there were concerns about how representative expert opinion would be:

‘There are some areas where … because there’s only one trial, or it’s only a controlled study … sort of were dismissed … because they weren’t sufficiently rigorous, but yet we were all allowed to pontificate and give our views, and get them in there’. (Healthcare professional 1, MH2)

Others considered evidence ‘of experience’ more positively:

‘Lack of evidence doesn’t mean to say that there’s … that’s a reason not to intervene at all. We do have to … use common sense and I think the same is true really to make sure that within the guidelines we’re not … taking lack of evidence as being negative evidence’. (Chair, AH2)

‘Where there was no evidence … so the recommendations were a little bit limited … we kind of had that freedom, as it were, to be able to draw on our own experience without needing this gold standard’. (Service user, MH2)

### The role of context and judgement in interpreting evidence

Two factors emerged that influenced the interpretation of evidence: the role of contextual factors (such as the type of intervention) and the role of judgement.

There were different views as to whether evidence should be considered differently according to the context:

‘There were discussions … saying there’s no evidence for X drug being useful, do not use it. Whereas, in the psychological treatments, there was no evidence for Y being a very good treatment, but … it didn’t actually say do not use it’. (Service user, MH2)

‘In the world of science two and two makes four much more simply than in the world of education’. (Professional member 2, PH1^f^)

The opposing view is illustrated with these examples:

‘You can’t pretend that evidence depends on the discipline. The evidence depends on the evidence’. (Chair, MH2)

Judgment by advisory group members was identified as an important factor in the interpretation of evidence:

‘[Group members]’ve got different areas of expertise; otherwise the NICE team could just get the evidence and write the guidance’. (Chair, PH2)

‘It’s pooling human judgments, isn’t it? This isn’t a process you can do by computer, or algorithmically’. (Healthcare professional 2, MH1)

Technical team members appeared to be slightly more cautious than advisory group members about using judgement in interpreting and using evidence:

‘For health economics … it’s not as clear-cut … if there’s not evidence available, we are allowed to go away and do models. And … if there’s not evidence available … then we’re allowed to make assumptions … which you would never do for any of the clinical questions’. (Health economist, AH1)

Group members considered that interpretation of evidence was dependent on context (Table 4). There did not appear to be differences by advisory group or membership apart from members of the technical team who, possibly related to their role, did not discuss whether interpretation of evidence was dependent on context.

**Table 4:** Evidence dependent versus not dependent on context

**Theme**	**Total**	**Advisory group**			**Members**			
		**Acute physical health**	**Mental health**	**Public health**	**Chair**	**Health profs.**	**Service users**	**Technical team**
Evidence dependent on context	14	6	5	3	2	6	6	0
Evidence not dependent context	3	0	3	0	2	1	0	0

### Who gets heard?

There were challenges for the group in managing the diverse range of opinions and vested interests of group members and ensuring that group members contributed equally to discussion and decision making. Service users’ input was valued and there were strategies to guard against tokenism. Fostering good relations was seen as important to the functioning of the group.

#### The challenges of diversity

Effectively accommodating the diverse opinions of group members was a challenge for all three advisory groups. These challenges included minimising professional protectionism:

‘Because they’re … all coming from different backgrounds, so some of them will try to defend what they’ve done the whole of their lives’. (Health Economist, MH1)

Another challenge resulting from the diversity of members was allowing time for them to bond:

‘There was real uncertainty within the group, and quite a wide divergence of opinion … it does take people quite a bit of time to, to sort of settle down in their thoughts … and make decisions’. (Chair, AH2)

Dealing with tensions arising where opinions differed was also seen as a challenge arising from diversity within the advisory group:

‘There were a lot of product champions in this field and … I thought that there may be difficulty in accommodating their views’. (Chair, MH1)

‘If I’m honest, there were tensions between grumpy academics and… like me, and a, sort of, annoyance at the repetition of certain issues, really’. (Professional member 1, PH2)

### Having a voice and having influence

Most members thought that discussion was dominated by some to the exclusion of other members, in particular service users:

‘I don’t remember many items we’ve discussed where I’ve heard a patient’s perspective sought or, or expressed’. (Healthcare professional 1, AH1)

‘It did strike me … that there are … some people who are more voluble and find it easier to get space on the floor than others’. (Healthcare professional 1, MH1)

There was also a perceived inequality between members’ contributions when it came to influencing decision making:

‘I think you would have to shout quite loud to say that you had equal influence’. (Service user, MH2)

‘I think there is a hierarchy of opinion-formers within the group… I think the hospital physicians would come nearer the top of it… Probably the … patient representatives [at the bottom]’. (Healthcare professional 2, AH1)

There were twice as many comments about there being unequal contributions of members to discussion and influence on subsequent decision making than comments describing equal contributions of members (Table 5). While this was the case among service users and health professionals, advisory group chairs were more likely to say there were equal contributions to discussion and decision making. This is of note given the responsibility of chairs to promote opportunities for all to contribute.

**Table 5:** Equality of contribution and influence

**Theme**	**Total**	**Advisory group**			**Members**			
		**Acute physical health**	**Mental health**	**Public health**	**Chair**	**Health profs.**	**Service users**	**Technical team**
Equal contribution/ influence	24	9	13	2	9	6	6	3
Unequal contribution/ influence	56	17	22	17	6	27	19	4

Unequal influence was seen by some as beneficial for decision making by preventing unfocussed discussion:

‘I think the group has some people … that will take the lead and … have strong arguments and convince the rest of them, because some of them like to be really vague and go back and forth all the time’. (Health economist, MH2)

### Managing vested interests

There was a general view that people would bring their own vested interests to the table, influenced by their experiences, their academic or professional speciality, or allegiance to particular ‘products’ (*e.g.*, types of treatment). Service users acknowledged this and considered these to be part of their role in the group:

‘I feel like I’ve gone into it very biased … I suppose that’s something to be expected. I mean, presumably, I mean … patient members, they are going to have a very biased, very solitary view in some respect and I have got one’. (Service user, MH1)

They also expected others in the group would be biased towards their own area of specialism:

‘I think everybody will be going in with their own individual bias, because nobody is impartial in this’. (Service user, MH1)

In view of this, the independent, neutral role of the NICE reviewers was seen as particularly important. The Chair of one advisory group, while acknowledging vested interests, did not consider that they impeded work of the advisory group.

‘There was very little partisanship. It wasn’t as though people said, you’ve got to do things this way, because I’m representing this constituency, as it were, and you can’t ignore them. There didn’t seem any of that happening, really’. (Chair, MH2)

The Chair of one advisory group saw the technical team as using scientific evidence to guard against biases bought to the group by ‘product champions’. When interviewed at the end of the process the Chair thought bias had been controlled in the group.

‘[The] systematic reviewer was trusted to be completely independent and just rely on the data. … [group members] respected that she was a genuinely independent investigator, who had no particular bias towards any of the treatments on offer’. (Chair, MH2)

Some advisory group members were faced with the problem of members evaluating their own research or practice:

‘I think you’ve got a problem actually when you, you’re basing a recommendation made on your own research. You, you’re just too emotionally, um, involved with it’. (Health Professional 1, AH2)

‘[Members of the advisory group] who were very proud, fiercely proud, of what they did and were doing it for the best reasons. Very hard to challenge best practice when somebody is so committed to best reasons’. (Professional Member 2, PH2)

The theme of bias appeared to be more common in the acute physical and mental health advisory groups compared with the public health group (Table 6). This may be related to the nature of the evidence appraised by these two groups, specifically considerations around the need to minimize bias. Utterances were coded as ‘bias’ if they referred to instances of or perceptions that the vested interests of advisory group members were apparent in discussions and decision making; utterances were coded ’no bias’ if advisory group members reported that members were not influenced by any particular allegiances.

**Table 6:** Bias

**Theme**	**Total**	**Advisory group**			**Members**			
		**Acute physical health**	**Mental health**	**Public health**	**Chair**	**Health profsCP**	**Service users**	**Technical team**
Bias	24	9	14	1	6	8	5	5
No bias	4	0	4	0	3	0	1	0

### The role of the service user

The general perception was that the services users did a good job and their contributions of challenging health professionals and adding their perspectives as recipients of healthcare were necessary and valued:

‘Obviously service users know far more about the experience of receiving that service than those who deliver it do’. (Healthcare professional 1, MH1)

‘To be reminded of what the person with [condition x] feels … you don’t get that unless you have a proper service user on the panel’. (Chair, MH2)

Some concern was expressed that the inclusion of service users was tokenistic and that this was evidenced by there being an average of only two service users compared to six to eight healthcare professional members serving on advisory groups:

‘Ah, now then. I think the, um, service-user representation is a bit tokenistic’. (Healthcare professional 2, MH1)

‘I think it says something about how much experts by experience are valued, because if we’re all equally valued, there’d be equal numbers of people’. (Healthcare professional 1, MH1)

Service users thought they carried less influence than other group members leading some to question why they had been invited to participate:

‘If I voiced an opinion what wasn’t evidence based, ah, and I think, let’s say for example, (clinician A) or (clinician B ) had opinion, their opinion, I think those would have had more sway than mine’. (Service User 1, AH2)

There appeared to be strategies in place to protect against the presence of service users as a ‘box-ticking’ exercise and to support them within the team:

‘[The Chair] makes a real effort to make sure that … he directly asks [the service user] things if he hasn’t been saying much and he tries to get, get a view’. (Health Professional 2, AP1)

### Group composition

The importance of fostering good social relationships was noted. Opportunities to socialise during the process, such as dinner before the meeting, facilitated more relaxed and productive discussion and led to the development of a more cohesive group:

‘I think there does need to be that team-building bit at the beginning’. (Professional Member 2, PH1)

‘The first few GDGs were a bit stilted as people were finding where, you know, how the group was going to operate, and I think now there’s a bit of banter and, um, you know, well, people just know each other better, so it, it works more, more smoothly’. (Healthcare professional 2, AH2)

This view appeared to be consistent across all groups.

### What is the process?

Group members spoke about the value of debate and the need for marshalling discussion to retain focus and avoid conflict. Most members were satisfied with the process and output.

### Managing discussion

Discussion was encouraged by some members as it was seen as a valuable part of resolving disagreement and necessary to consider a variety of perspectives in order to improve the quality of recommendations:

‘I do genuinely believe that the more debate the better the findings’. (Professional Member 1, PH1)

‘It would be a terrible shame if you had a group which did have no dissenting voices at all. It would be very dull and probably produce very unchallenging … results at the end’. (Chair, AH2)

Discussion appeared to be curtailed for two reasons: to stop unfocussed time-consuming debate (task-focused chairing) and to minimize the potential for conflict between group members (emotion-focused chairing). One strategy used by the Chair and co-Chair in one advisory group to keep discussion on track was to actively steer the group away from conflict:

‘He [co-Chair] is very skilled indeed at, um, bringing a group round to an opinion, which represents consensus and steering them away, similarly, from areas where they might disagree’. (Chair, MH2)

Another strategy to curtail discussion was to steer members towards consensus:

‘It can be the case that we’d spend a hell of a lot of time discussing, but actually not pushing on with the task … we just needed to be pushed, and when pushed, we came up with the goods’. (Healthcare professional 1, MH2)

But these strategies also created some dissatisfaction about the lack of debate:

‘By the time we’d got to the stage where actually we were comfortable enough as a group to actually have some of those very frank discussions … the NICE guideline machine … took over, and we became … focused on the … nicety of text’. (Healthcare professional 1, MH2)

Members suggested that the lack of debate would have a negative impact on decision making and the quality of guidance developed:

‘It all seemed a little bit tame … which is all right, you know, I mean, who wants to go to work and have fights—but it did just, it surprised me, and maybe it was a little bit ineffectual because of that’. (Service user, MH2)

### Satisfaction with process and output

Members expressed satisfaction with the process of developing the guidelines.

‘I think it’s gone remarkably well ah, considering how the, how the groups meet up, and how sort of limited work, is actually done within, within the meetings’. (Service user, MH2)

‘Well, this is my first encounter with a NICE guideline group, and I have to say it’s been a tremendously uplifting experience, because it’s gone extremely well, in my view, and it’s been very smooth by and large’. (Chair, MH2)

Some members thought there was not enough time to complete recommendations, with a sense of being rushed towards the end:

‘The actual wording of the recommendations struck me as a little bit rushed and a bit of a free for all towards the end’. (Healthcare professional 2, MH2)

‘There was a bit of steamrollering going on. We were, more or less, accepting things, because there was no time left to discuss it properly’. (Chair, MH2)

While other members were more pragmatic:

‘There’s a finite time you can spend on each recommendation, arguing over one preposition, isn’t there? You’ve got to be very pragmatic about it, and say, actually that’s good enough’. (Healthcare professional 2, MH2)

Factors that influenced satisfaction with the process included small group work. This was valued as a way of working effectively and allowing equality of input:

‘If you’ve got a small group people interact a lot better’. (Health economist, AH2)

‘These smaller group working sessions … [ARE] … an opportunity for people who … prefer the smaller group to, to be able to get their views across’. (Community Member 2, PH1)

‘They (ADVISORY GROUP MEMBERS) tend to be more productive in their small groups’. (Chair, PH1)

The guidance development process was considered to benefit from a transparency of process:

‘For me it’s transparent, validated, rigorous processes’. (Professional Member 1, PH2)

‘One of the nice things which, I think, is central to the NICE process is transparency. And I think, I think we were very transparent all the way through’. (Chair, MH2).

## Discussion

### How do findings compare with existing literature?

Evidence from observational and experimental studies suggest that the same evidence can be interpreted differently by different groups [15] and that experience, composition of specialisms, and professional status within groups influence how recommendations are formed [12-14]. This study lends further support to these findings and extends them by giving a voice to group members that affords a tone and level of detail not possible with other methods.

Variation in group members’ views of what evidence is identified in this study may go some way to explaining observations in previous research of different groups interpreting the same evidence differently [15].

Advisory group members in this study spoke about the vested interests they and other members bring to the table. This is similar to previous work reporting how professional experiences and beliefs hold sway over evidence in influencing recommendations [12,13].

An inequality in contributions to discussion within advisory groups was observed in this study. The notion of a ‘hierarchy of opinion formers’ aligns with previous findings that professional status was a key factor in the recommendation formation process [14].

Themes emerging around the role of the service user echoes aspects of previous work reporting barriers and facilitators to user involvement. Some group members considered service users to be under-represented in advisory groups; previous research has reported this as a key barrier to service user involvement. The role of the chair in encouraging contributions from service users to discussion and decision making observed in this study echoes previous work reporting this as a key facilitator in user involvement. Working in smaller groups, considered by service users in this study to promote effective working, has also been reported as a facilitator to service user involvement [16].

The larger number of second interviews could be attributable to study researchers attending meetings during the life of the guideline development group and engaging and recruiting group members to the study. Future research in this area might consider a more proactive recruitment strategy at the start of the life of an advisory group to engage and recruit group members.

### Implications for practice

The main implications for practice from this study relate to differences of opinion on fundamental issues.

Extensive guidance exists and is made available to advisory group members on the function of recommendations, evaluating different kinds of evidence and what kinds of evidence to consider when forming recommendations. Despite this, the range of opinions in these areas voiced by advisory group members in this study highlight important issues. These differences may result in unnecessary, protracted debate on evidence and the nature of recommendations made. To optimize the extensive time and financial investment in guidance development, it is important to keep discussion as efficient as possible. One way of promoting this would be to ensure advisory group members are aware of the guidance relating to evidence and the purpose of guidelines.

While the aim is to achieve a cost-effective process of guideline development; there appear to be some activities warranting investment to deliver an effective service. In this study, advisory group members considered these activities to be allowing sufficient opportunity for members to bond, *i.e.*, to undergo the form, storm, norm, perform phases of group development [17] and time to work in smaller groups.

In this study, we have identified differences between group members in views and perceived tensions. This raises two research questions: How do the different views and tensions expressed here manifest themselves in group processes? Given the tensions and discrepancies in viewpoints, how is it that groups work well and in the end members appear to express satisfaction with both process and output? We would hope that the findings from this study will be picked up by those involved in the process of reflecting on and attempting to improve the practice of health service advisory groups.

## Conclusions

By eliciting the views of those directly involved in developing evidence-based guidelines for medicine we have built on the experimental and observational work of others. We have been able to confirm previous observations of the role factors including professional status in influencing groups’ discussions and decision making.

This study highlights that evidence doesn’t form recommendations itself but requires human judgement—the strength of advisory groups appears to lie in the diversity of opinion judging and debating evidence used to form recommendations that comprise evidence-based guidelines.

The data show a plurality of viewpoints in the interviewees’ responses. This is not in itself surprising given the explicit commitment in NICE’s process and methods to the value of deliberative democracy which actively encourages and nurtures such pluralism. What then is all the more remarkable is that consensus is the endpoint. In the three groups studied, when the guidance was published there were no members who wanted to dissociate themselves from the recommendations and in our study most participants expressed satisfaction with the process and outcome. This may reflect the social component of the group process as individuals come to identify themselves as group members. Getting to this position though, as the data show, is sometimes a painful process. In the end, it seems that members come to acknowledge that evidence (however it is defined) alone does not determine recommendations and that other broader considerations come into play. These come into play through a mixture of individual and group processes, some of which are described here.

### What this study adds to the evidence

In order to improve any process, the first step is to understand it. The first step in understanding is to document what is happening. We believe this study is the first step in documenting the process of recommendation formation in order to understand and inform any future improvement.

## Endnotes

^a^Until April 1st 2013 NICE was known as the National Institute for Health and Clinical Excellence;

^b^We acknowledge that other terms such as ‘patient representatives’, ‘consumer advocates’, ‘consumers’, ‘users’ are used elsewhere used to describe, patients, carers and family members as well as representatives of charities and patient organizations. For the purposes if this report they will be referred to as ‘service users.

^c^MH1: mental health advisory group, time 1 interview; MH2: mental health advisory group, time 2 interview.

^d^Ellipses indicate where sections of quotes have been omitted deliberately.

^e^AH1: acute physical health advisory group time 1 interview; AH2: acute physical health advisory group time 2 interview.

^f^PH1: public health advisory group time 1 interview; PH2: public health advisory group time 2 interview.

## Competing interests

SM is an associate editor of Implementation Science and a member of NICE’s Public Health Interventions Advisory Committee. MK has been responsible for the methods and processes manuals developed by NICE to inform its public health work and is Director of the Centre of Public Health at NICE.

## Authors’ contributions

SM conceived and designed the study. LA and SM analysed the data. LA, JS, MK, and SM interpreted the results. LA and SM drafted the manuscript. JS, MK, and SM contributed to revisions of the manuscript. All authors read and approved the final manuscript.

## Supplementary Material

Additional file 1 Interview schedules. Click here for file

## References

[B1] Daniels N Just health: meeting health needs fairly 2008 Cambridge: Cambridge University Press

[B2] Daniels N Sabin JE Setting limits fairly: learning to share resources for health 2008 2 Oxford: Oxford University press

[B3] Eccles MP Grimshaw JM Shekelle P Schunemann HJ Woolf S Developing clinical practice guidelines: target audiences, identifying topics for guidelines, guideline group composition and functioning and conflicts of interest Implement Sci 2012 7 1 60 10.1186/1748-5908-7-60 22762776 PMC3523009

[B4] Gardner B Davidson R McAteer J Michie S A method for studying decision-making by guideline development groups Implement Sci 2009 4 48 10.1186/1748-5908-4-48 19656366 PMC2731071

[B5] Hopthrow T Feder G Michie S The role of group decision making processes in the creation of clinical guidelines Int Rev Psychiatry 2011 23 358 364 10.3109/09540261.2011.606539 22026492

[B6] Michie S Berentson-Shaw J Pilling S Dieppe P Raine R Cluzeau F Alderson P Ellis S Feder G Turning evidence into recommendations: protocol of a study guideline development groups Implement Sci 2007 2 29 10.1186/1748-5908-2-29 17803806 PMC2031892

[B7] Kelly M Morgan A Ellis S Younger T Huntley J Swann C Evidence based public health: a review of the experience of the National Institute of Health and Clinical Excellence (NICE) of developing public health guidance in England Soc Sci Med 2010 71 6 1056 1062 10.1016/j.socscimed.2010.06.032 20678836

[B8] Kelly MP Moore T The judgement process in evidence based medicine and health technology assessment Social Theory and Health 2012 10 1 19 10.1057/sth.2011.21 23226973 PMC3500844

[B9] Raine R Sanderson C Black N Developing clinical guidelines: a challenge to current methods BMJ 2005 17 331 631 7517 16166137 10.1136/bmj.331.7517.631 PMC1215565

[B10] Moreira T May C Mason J Eccles M A new method of analysis enabled a better understanding of clinical practice guideline development processes J Clin Epidemiol 2006 59 11 1199 1206 10.1016/j.jclinepi.2005.08.021 17027431

[B11] Burgers JS Bailey JV Klazinga NS Van der Bij AK Grol R Feder G Inside guidelines: comparative analysis of recommendations and evidence in diabetes guidelines from 13 countries Diabetes Care 2002 25 11 1933 1939 10.2337/diacare.25.11.1933 12401735

[B12] Raine R Sanderson C Hutchings A Carter S Larkin K Black N An experimental study of determinants of group judgments in clinical guideline development Lancet 2004 364 9432 429 437 10.1016/S0140-6736(04)16766-4 15288741

[B13] Hutchings A Raine R A systematic review of factors affecting the judgments produced by formal consensus development methods in health care J Health Serv Res Policy 2006 11 3 172 179 10.1258/135581906777641659 16824265

[B14] Pagliari C Grimshaw J Impact of group structure and process on multidisciplinary evidence-based guideline development: an observational study J Eval Clin Pract 2002 8 2 145 153 10.1046/j.1365-2753.2002.00333.x 12180363

[B15] Hemingway H Chen R Junghans C Timmis A Eldridge S Black N Shekelle P Feder G Appropriateness criteria for coronary angiography in angina: reliability and validity Ann Intern Med 2008 149 4 221 231 10.7326/0003-4819-149-4-200808190-00003 18711152

[B16] Legare F Boivin A van der Weijden T Pakenham C Burgers J Legare J St-Jacques S Gagnon S Patient and public involvement in clinical practice guidelines: a knowledge synthesis of existing programs Med Decis Making 2011 31 6 E45 E74 10.1177/0272989X11424401 21959267

[B17] Tuckman B Developmental sequence in small groups Psychol Bull 1965 63 384 399 14314073 10.1037/h0022100

