# Supplementary material for: The process of developing evidence-based guidance in medicine and public health: a qualitative study of views from the inside
Source: Implement Sci. 2013 Sep 4;8:101. doi: 10.1186/1748-5908-8-101 (PMC3846576; doi:10.1186/1748-5908-8-101)
Supplement: Additional file 1 — Interview schedules. [file 1748-5908-8-101-S1.docx]

**Additional file 1: Interview schedules**

**First interview schedule**

1. Can you describe the process through which recommendations will be decided in your GDG?
2. Has this process been clearly communicated from the beginning?
3. What factors would you take into consideration when making or deciding on a recommendation?
4. Do you think that other members of the GDG might take different factors into account when making a recommendation? [Prompt: What would these be?]
5. What do you understand the word ‘evidence’ to mean in the GD process?
6. How will you evaluate the importance of different types of ‘evidence’?
7. Do you think your understanding and use of the word ‘evidence’ might be different from other members of the group? [Follow-up: Could these differences potentially influence the recommendations to be developed by the group?]
8. Has there been any tension or conflict or disagreement between people so far? [Follow-up: Can you describe a recent incident where this happened? How are decisions made when there are difficulties between people? Has it had any effect on you? Has it affected the way you contribute to the group?]
9. What do you think the purpose of the recommendations is?
10. Do you think the evidence that is referred to may result in recommendations that would be impossible or very difficult to implement? [Prompt: Why?]
11. Are you aware of any existing relationships between group members which you feel may impact on the guideline process?
12. Do you notice any emerging dynamics or patterns between individuals so far?
13. Do you think there is anyone missing or under-represented in the group?
14. Is there anything that surprised you about the group’s set-up?

**Second interview schedule**

1. Over the period of guideline development, what were your views on the progress of the PSHE group?

[Related: Did your experience of the PDG meet your expectations (if you had any); What has the group achieved?]

1. Can you describe the processes through which recommendations were decided in your group (PDG)?
2. Do you think the processes were clearly communicated from the beginning?
3. What do you think the purpose of the recommendations is?
4. What factors did you take into consideration when making or deciding on a recommendation?
5. Do you think that other members of the PDG took different factors into account when forming recommendation? [Prompt: What do you think these were?]
6. What do you understand the word ‘evidence’ to mean in the guideline development process?
7. Do you think that your idea of what constitutes evidence changed through the process of guideline development?
8. How did you evaluate the importance of different types of ‘evidence’?
9. Do you think your understanding and use of the word ‘evidence’ was different from other members of the group? [Follow-up: Did these differences potentially influence the recommendations to be developed by the group?]
10. Do you think the group members interacted effectively with one another? Were there any emerging dynamics or patterns that you noticed between individuals? [Prompts: How could the dynamics within the group have been improved? Why might this change be an improvement?]
11. Did you notice any tension or conflict or disagreement between people within the group? [Follow-up: Can you describe a particular incident where this happened? How were decisions made when there are difficulties between people? Did this have any affect on you? Did it affect the way you contribute to the group?]
12. Were you aware of any existing relationships between group members which you feel may impacted on the guideline process?
13. Do you think there was anyone missing or under-represented in the group?
14. Was there anything that surprised you about the group’s set-up?
15. Did the group set up change over the course of the guideline development?
16. What influence did this have on guideline development?
17. Did exposure to other group members’ opinions change your outlook in any way?
18. What effect do you think external factors had on the development of the guidelines? The dynamics within the group? Other group members? On your own input? [May prompt with specific aspects about change in Government to coalition and associated changes]
19. Do you think the process achieved its aims? Are you happy with the outcome?
